# Gold Nanocomposite Contact Lenses for Color Blindness Management

**DOI:** 10.1021/acsnano.0c09657

**Published:** 2021-02-11

**Authors:** Ahmed E. Salih, Mohamed Elsherif, Fahad Alam, Ali K. Yetisen, Haider Butt

**Affiliations:** † Department of Mechanical Engineering, Khalifa University, Abu Dhabi, United Arab Emirates; ‡ Department of Chemical Engineering, Imperial College London, London SW7 2AZ, United Kingdom

**Keywords:** nanocomposites, color blindness, wearables, contact lenses, biomaterials

## Abstract

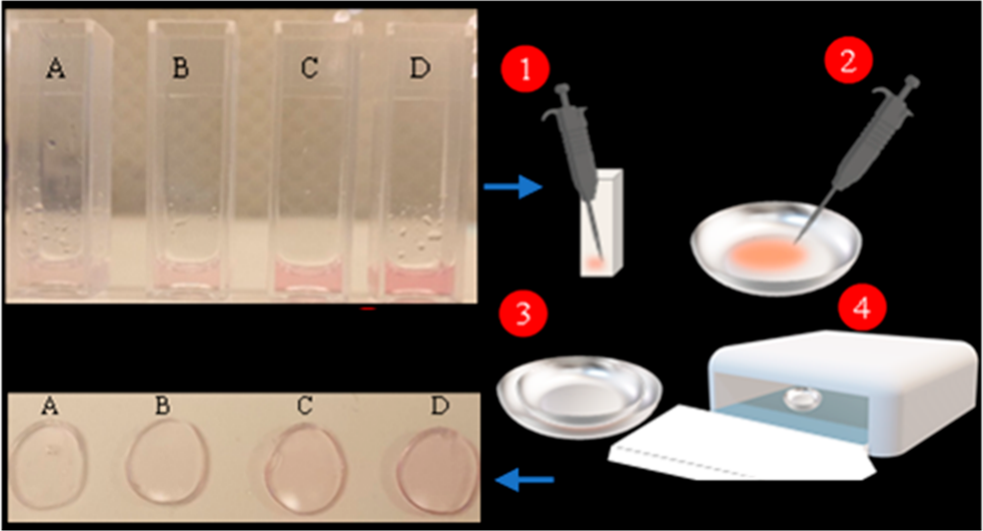

Color vision deficiency (CVD) is an ocular congenital disorder that affects 8% of males and 0.5% of females. The most prevalent form of color vision deficiency (color blindness) affects protans and deutans and is more commonly known as “red–green color blindness”. Since there is no cure for this disorder, CVD patients opt for wearables that aid in enhancing their color perception. The most common wearable used by CVD patients is a form of tinted glass/lens. Those glasses filter out the problematic wavelengths (540–580 nm) for the red–green CVD patients using organic dyes. However, few studies have addressed the fabrication of contact lenses for color vision deficiency, and several problems related to their effectiveness and toxicity were reported. In this study, gold nanoparticles are integrated into contact lens material, thus forming nanocomposite contact lenses targeted for red–green CVD application. Three distinct sets of nanoparticles were characterized and incorporated with the hydrogel material of the lenses (pHEMA), and their resulting optical and material properties were assessed. The transmission spectra of the developed nanocomposite lenses were analogous to those of the commercial CVD wearables, and their water retention and wettability capabilities were superior to those in some of the commercially available contact lenses used for cosmetic/vision correction purposes. Hence, this work demonstrates the potential of gold nanocomposite lenses in CVD management and, more generally, color filtering applications.

Color vision deficiency (CVD), more commonly known as color blindness, is an inherited ocular disorder that limits sufferers’ ability to distinguish between specific colors; the latter depends on the disorder type and its severity.^1−3^ It limits the range of activities or chores the patients can perform. For instance, CVD patients are restricted from working in fields like the military, aviation, and certain medical fields since color recognition is critical in these occupations.^4,5^ Further, humans’ eyes perceive colors *via* the photoreceptor cones located at the back of the eyes (Figure 1a). There are three types of photoreceptor cones, namely, short (S)-cone, medium (M)-cone, and long (L)-cone. These cones are also referred to by the colors that they are most sensitive toward. Indeed, blue, green, and red photoreceptor cones refer to the S-cone, M-cone, and L-cone, respectively.^3,6,7^ Furthermore, depending on the wavelength of the incoming light, the cones are activated at different levels, and the color perceived by the eye is the combination of the signals from the three cones.

**Figure 1:**
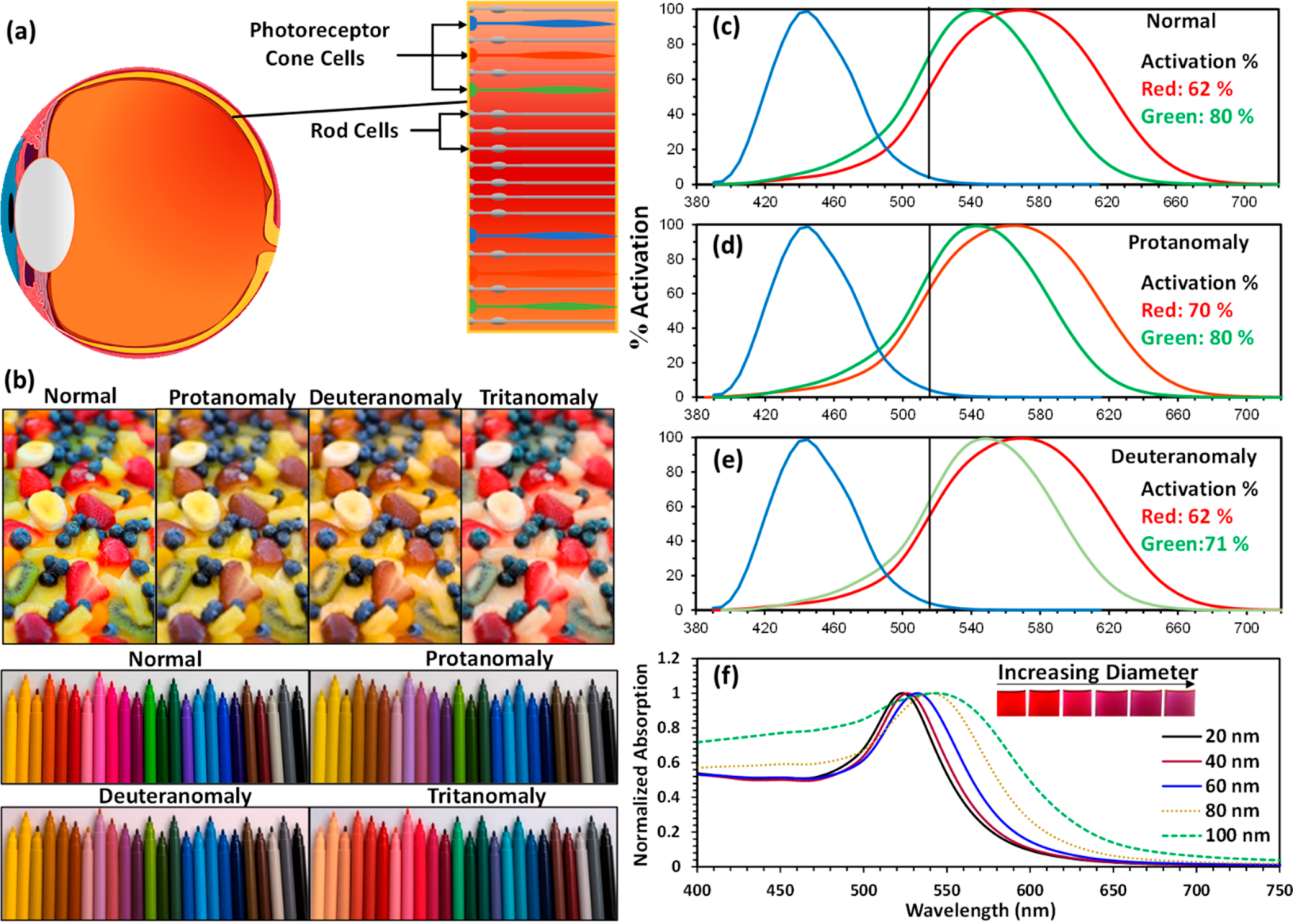
Visual perception in color vision deficiency. (a) Photoreceptor cone and rod cells inside the eye. (b) Images of colored materials as seen by normal color vision and different types of CVDs. Photoreceptor cells’ activation percentage at 520 nm for (c) normal, (d) protan, and (e) deutan. (f) Mie theory simulated absorption spectra of gold nanoparticles as a function of their diameters.

People with normal color vision are referred to as trichromats.^3^ On the other hand, CVD patients are usually described by their deficiency type. There are three CVD categories corresponding to the defect types, namely, anomalous trichromacy (faulty photoreceptor cone), dichromacy (missing photoreceptor cone), and monochromacy (at least two missing photoreceptor cones).^7^ Within each of the former two categories, the CVD is described based on the defected photoreceptor cone. Patients who have deficiencies in the blue, green, and red cones are referred to as tritans, deutans, and protans, respectively. More specifically, in anomalous trichromacy, the CVD types are referred to as tritanomaly, deuteranomaly, and protanomaly, and in dichromatism, they are tritanopia, deuteranopia, and protanopia.^3,8^ However, monochromacy is divided into achromatopsia (total color vision loss) and blue cone monochromacy (missing red and green cones); monochromacy is the rarest form of CVD.^3,6,8^ Furthermore, deutans and protans are commonly identified as having red–green color blindness. Moreover, red–green color blindness constitutes 95% of all CVDs, making it the most prevalent form.^3,9^

CVD sufferers are diagnosed through various methods, but the simplest and most commonly used is the Ishihara testing plates.^10^ The Ishihara test is a pseudoachromatic test which utilizes differences in color and contrast to identify whether an individual is color blind or not. Each plate consists of dots having different colors, which forms a specific number; CVD patients struggle to differentiate between the colors in the plate. Consequently, they fail in naming the number in the plate. The downfall of the Ishihara test is that it can only identify patients with red–green color blindness.^10,11^ However, tests like Richard HRR can diagnose protans, deutans, and tritans. Richard HRR is another pseudoachromatic test in which symbols are used instead of numbers. Unlike the Ishihara test, the Richard HRR test can diagnose the CVD type and its severity.^10,12,13^

In recent years, extensive research on the treatment of CVD in non-human primates through gene therapy showed promising results; however, the latter is yet to be applied on humans.^14,15^ Hence, most CVD sufferers rely on wearables to manage the difficulties endured in their day-to-day tasks. The most common wearable is a form of tinted glass/lens.^16,17^ Furthermore, the principle behind using the filters was introduced by Seebeck who claimed that when red and green filters are placed successively, protans and deutans are able to differentiate between shades of indistinguishable colors.^17^ This was later developed into lenses and glasses by different companies; of these companies, Enchroma is the most well-known for providing tinted glasses.^18^ Moreover, as tested by Enchroma and other companies, CVD corrective glasses have experimentally shown their efficacy in improving sufferers’ color contrast and, thus, perception. These glasses are customized, and they filter out a range of problematic wavelengths, based on the patient’s CVD. The range of these wavelengths is often 520–580 nm (for red–green crossover) and 440–500 nm (for blue–green crossover).^19^ For lenses, companies like Chromagen have developed red contact lenses to aid CVD patients, but their reported effectiveness varied among tested patients.^20^ More recently, organic Atto dyes have been used to alter the colors of contact lenses. However, the stability of the dyes within the contact lenses is yet to be improved. In fact, it was shown that the dyes’ effectiveness was reduced by 40% after 1 day due to their leakage from the lenses.^21,22^ Also lately, smart glasses developed by companies, like Google, have been incorporated into CVD research.^23,24^ Researchers used these wearables to actively filter and recolor the vision of the patients using image processing algorithms; however, the drawback of such glasses is their bulk size, which makes them impractical for daily use.

Research on CVD management techniques has shown the ineffectiveness of dyed contact lenses as those have leaching and toxicity problems. In this work, gold nanoparticles (GNPs) are incorporated within contact lenses to aid red–green CVD patients. Noble metal nanoparticles (NPs), particularly gold and silver, have excellent electrical and optical properties, making them suitable for various biomedical applications like molecular imaging, targeted drug delivery, and biosensor fabrication.^25−27^ Moreover, gold and silver nanoparticles’ surface plasmon resonance (SPR) facilitates their excellent light absorption and scattering properties.^28^ SPR results from the motion of the nanoparticles’ conduction electrons, which are roaming freely, up until their interaction with the incident light. The electrons then oscillate as a result of the electric field produced by the incident light. Nanoparticles have specific plasmonic frequencies depending on their morphology, namely, shape, solvent, and size. Furthermore, SPR occurs when the incident light’s frequency matches the plasmonic frequency of the nanoparticles.^28,29^

Gold nanocomposites (GNCs) have been utilized for a variety of optical applications, yet the early 19th century was when gold nanoparticles were used as a colorizing agent for glass in 1802. Nonetheless, gold ruby (red) glasses are still commercially manufactured.^30^ Moreover, silicone hydrogel contact lenses were exposed to gold and silver nanoparticle solutions in order to alter their optical properties. Lenses were first submerged in the NP solutions, then removed, and washed using DI water to remove nonabsorbed particles from the surface. Transmission and absorption spectra of the lenses were similar to those of the original NP solution. The silver- and gold-doped contact lenses were developed to aid patients suffering from retinal or ocular disorders that does not allow them to work properly in bright light environments.^31^ Also, gold nanoparticles were added into a polyethylene matrix using solution casting for color filtering applications.^32^ The orientation of the nanocomposites was shifted by uniaxial drawing, and its effect on the absorption spectra was studied. The absorption spectrum was shown to depend mainly on the polarization direction of the incoming light. The nanocomposites appeared red and blue when light was polarized perpendicular and parallel to the drawing axis, respectively. Authors report that this anisotropy was due to the morphology of the nanocomposites. Such nanocomposites can be used as polarization sensitive color filters.^32^

Here, GNPs were embedded within *in situ* synthesized contact lenses to filter out the range of optical wavelengths, at which CVD patients struggle to distinguish between specific colors. In characterizing the nanoparticles, their morphology was first studied using transmission electron microscopy (TEM), and their colloidal optical transmission was obtained using a UV/vis spectrophotometer. Also, since the refractive index of the medium was previously shown to alter the optical properties of the nanoparticles,^33^ nanoparticles were immersed in solvents having a different refractive index to test the effect of the latter on the transmission dip and bandwidth. Subsequent to obtaining the hydrogel nanocomposite lens, its transmission spectrum was recorded, and the distribution of the nanoparticles within the hydrogel was observed under scanning electron microscopy (SEM). In addition to the latter, the effect of the nanoparticles on the lenses’ water content and hydration contact angle was also studied. It is worth noting that the nanoparticles defy the limitations presented by the dyes, for they are both biocompatible and stable within the hydrogel matrix. Therefore, if the developed hydrogel nanocomposites showed superior optical and material properties, nanoparticles could potentially replace dyes as the filtering mechanism for CVD corrective contact lenses.

## Results and Discussion

**Figure 2:**
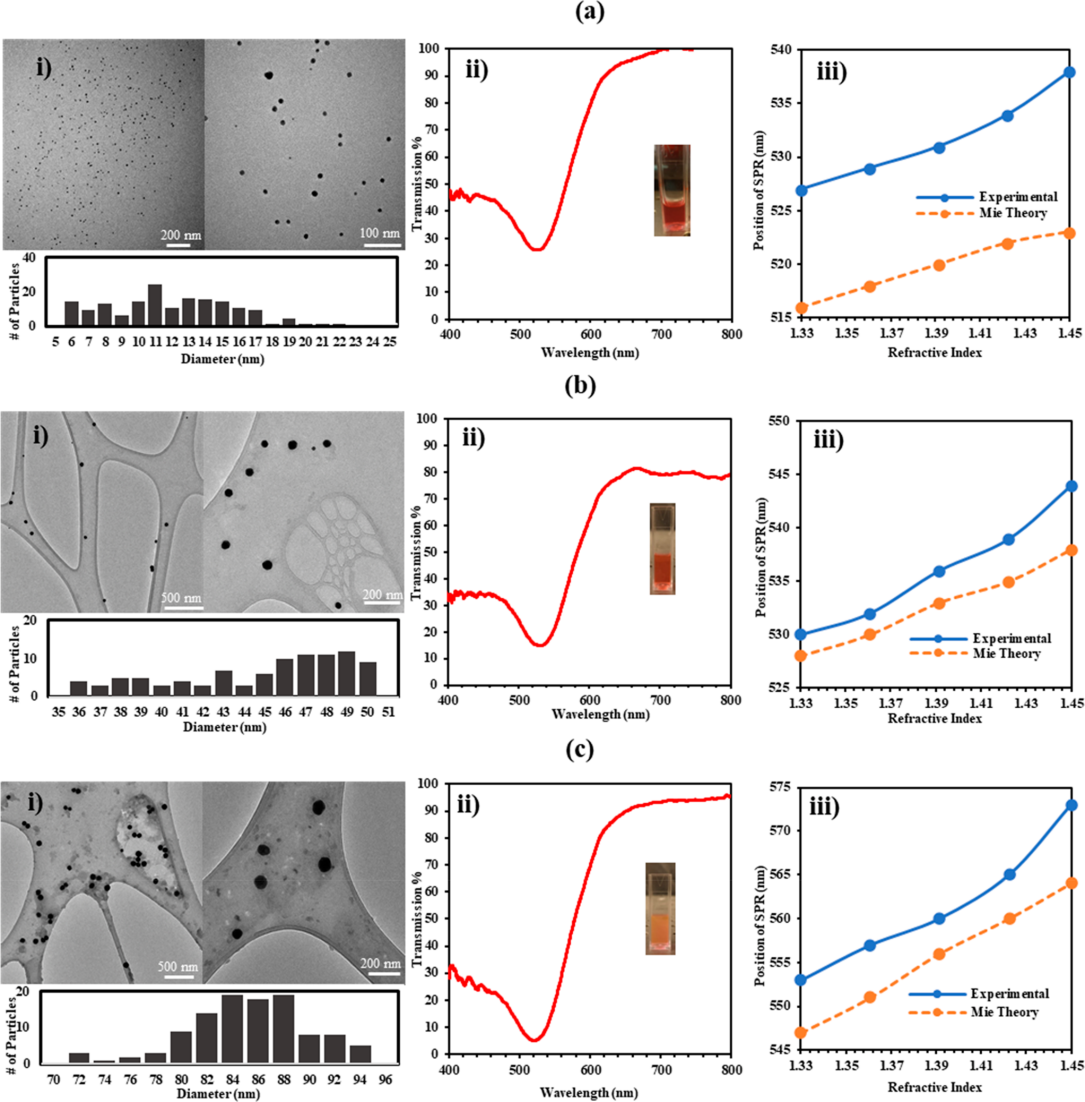
Prepolymerization characterization of the (a) 12 nm GNPs, (b) 40 nm GNPs, and (c) 80 nm GNPs: (i) TEM micrographs of the nanoparticles with their size distribution histograms; (ii) transmission spectrum of the nanoparticles in their solution; (iii) effect of varying the nanoparticles solution’s refractive index on the position of the surface plasmon resonance both experimentally and as predicted by the Mie theory.

The prepolymerization characterization is shown in Figure 2. The characterization was done for the three sets of nanoparticles outlined in the Methods section. The diameter of the three nanoparticle sets was 12.73 ± 4.01, 44.31 ± 4.17, and 85.82 ± 6.57 nm, in respective size order, as shown through their size histograms. The standard deviations of the diameters for both the 40 and 12 nm sets of GNPs were relatively high as they constituted almost 10 and 30%, respectively, whereas the 80 nm was less polydisperse as its standard deviation was only 6.5% of the nanoparticles’ diameter. Nevertheless, the TEM images (Figure 2) show that all of the gold nanoparticles were evenly distributed, and there were no signs of aggregation or agglomeration. The transmission spectra are shown in the second part of the figure (Figure 2ii). The transmission dip (surface plasmon wavelength) for the three GNPs occurred at 527, 530, and 556 nm, in the respective order of their sizes. Moreover, the full width at half-maximum (fwhm) measures the transmission bandwidth, and the recorded values for the three nanoparticle sets were 34, 45, and 47 nm, respectively. As expected, the increase in size of the nanoparticles increased the surface plasmon wavelength and the transmission dip’s bandwidth. The latter is caused by a shift in the energy levels required to displace the conduction electrons of the nanoparticles, which is also outlined through Mie theory. The Mie theory is an analytical solution to the Maxwell’s equations, and it explains the extinction caused by scattering and absorption behaviors of the nanoparticles.

Further, the transmission spectra of the three NPs were recorded at their initial solution of water, which has a refractive index of 1.33. However, it is vital to show how the nanoparticles are affected in case the refractive index of their solutions changes. More importantly, this characterization is critical to demonstrate how the transmission of the nanoparticles will be altered if they are mixed with polymers that have a refractive index different than that of HEMA. For instance, some of the commonly used polymeric materials in contact lenses are PMMA, PDMS, PVA, and polyacrylamide, and their refractive indices are 1.485, 1.40, 1.47, and 1.50, respectively. In addition, the experimental observations were supplemented with the predictions from Mie theory. In Mie theory, the refractive index of the nanoparticles’ medium and the size of the nanoparticles are the main parameters affecting the scattering and absorption profiles of the nanoparticles. Although the theory does not account for the particles’ interactions among themselves, it provides an adequate justification for the absorption/transmission spectrum of the nanoparticles at a given size and in a medium of a specific refractive index. Figure 2iii shows the effect of varying the refractive indices of the solutions of three sets of nanoparticles on the position of the surface plasmon wavelength. Evidently, the increase in refractive index red-shifted the SPR’s position (wavelength), which occurred mainly due to two reasons. The apparent first effect is that the change in refractive index induces a change in the light wavelength in the vicinity of the nanoparticles. The second effect is related to the polarization of the dielectric medium. Due to the SPR, charge accumulation at the vicinity of the NPs creates an electric field (other than that of the incident light). This charge is transferred to the edges of the medium (polarization), and hence, partial charge compensation occurs, which reduces the effective charge near the NPs. Moreover, increasing the dielectric function of the medium increases the polarization effect, which reduces the charge on the NPs’ surface. This causes the restoring force and frequency to decrease, hence, the increase in wavelength. This is analogous to the change in restoring force of an oscillator.^28^ Yet, the behavior of all NPs was not similar. For instance, the experimental and Mie theory results for the 12 and 40 GNPs were similar in trend, with the steepness of both plots being analogous, while the Mie theory curve for the 80 nm gold nanoparticles was dissimilar to the experimental curve. This indicates that, since the Mie theory predicts the absorption of a single particle, its estimates deviate more from the collective nanoparticles’ actual transmission as the size of the particles increases. The deviation might also be due to the constants used in simulating the Mie theory, as it was reported previously that the accuracy of the constants significantly affects the agreement with the experiment.^33^ Here, the Johnson & Christy constants were utilized. Moreover, the range of experimental SPR shift increases with the size increase, as well. In fact, the SPR ranges of 12, 44, and 85 nm gold nanoparticles were 11, 14, and 21 nm, respectively. This is probably due to the size difference in NP formed clusters. In other words, when bigger nanoparticles cluster, they shift the SPR even more than the shift induced by smaller NP coalescence.

**Figure 3:**
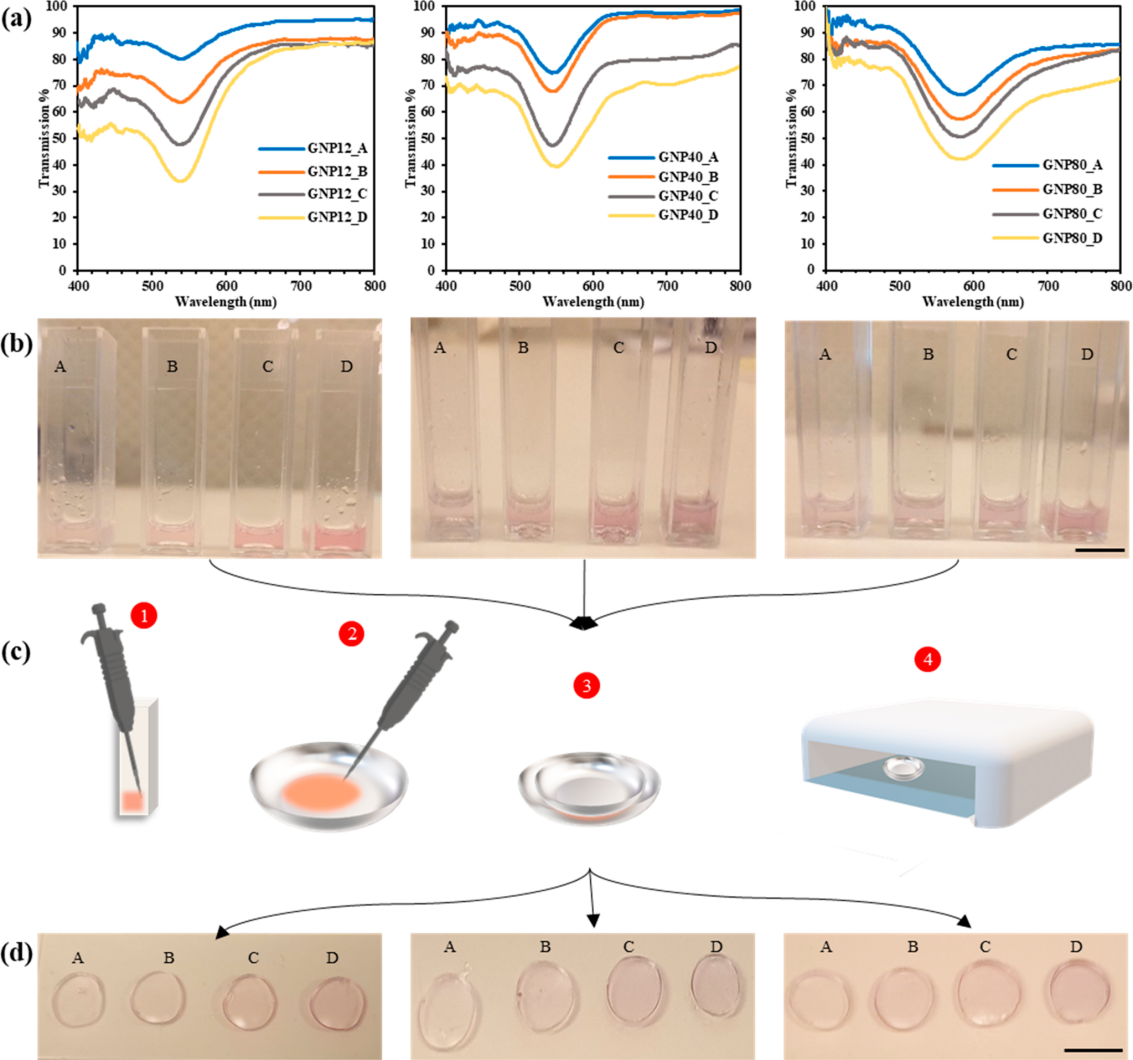
Polymerized 12 nm GNCs, 40 nm GNCs, and 80 nm GNCs (from left to right): (a) transmission spectra of the polymerized nanocomposites; (b) solutions of the nanocomposites prior to polymerization (scale: 10 mm); (c) steps carried out in polymerizing the solutions and obtaining the nanocomposite lenses; (d) polymerized nanocomposite lenses at different concentrations (scale: 10 mm). Note that A and D have the lowest and highest concentration of added nanoparticles, respectively.

The transmission spectra of the developed nanocomposites along with their images before and after polymerization are shown in Figure 3. For each of the three sets of nanoparticles, four different volumetric concentrations were added to the hydrogel solution, where A and D denote the samples with the lowest and highest NP concentrations, respectively. This was done to investigate the effect of NP addition on the transmission spectrum of the developed nanocomposite lens. The first apparent and clear distinction is the increased light blockage rate at higher concentrations. For instance, at the transmission dip, the four distinct 12 nm gold nanocomposites, ordered by their increasing concentration levels, blocked 15, 27, 42, and 58% of the incoming light. Similarly, the 40 nm gold nanocomposites, blocked 25, 32, 52, and 61%, respectively. Another characteristic that can be analyzed from the transmission spectra is the position of SPR wavelength (transmission dip wavelength), which was not altered as a result of the NPs’ addition to the hydrogel and remained at its initial value. The latter was noted for all three nanocomposite sets (Figure 3a), indicating that further light blockage can be achieved by increasing the amount of NPs within the lens without affecting the peak filtered wavelength.

Nevertheless, the transmission bandwidth or fwhm was affected by the nanoparticles’ addition. The increase in nanoparticles’ concentration generally increased the fwhm of the nanocomposites’ transmission. This is noted in both the 40 and 80 nm GNCs. Yet, initially in the 40 nm GNC, the increase in nanoparticle concentration caused the fwhm to decrease, indicating that the nanoparticles were well-dispersed in the lens. Further addition of the nanoparticles caused the bandwidth of the transmission dip to increase, which suggests that some nanoparticles became aggregated. The latter implies that the NP concentration of sample B was the optimum concentration at which light was effectively blocked with reduction in the transmission bandwidth. Such an optimum point was not observed in the nanocomposites of the 80 nm set; their fwhm was also severely affected as the minimum fwhm achieved was 110 nm, which is almost a 2-fold increase from the 40 nm set. The increase in concentration caused the transmission dip to widen even more particularly at the highest concentration, at which the fwhm was 129 nm. Transmission bandwidths, like those of the 80 nm nanocomposites, are highly undesirable for color-deficient patients as they filter out much of the colors that they can easily distinguish. It is worth noting that the fwhm of the 80 nm gold nanoparticles in their solution was 52 nm. The discrepancies in the fwhm of the 80 nm nanocomposites could have been due to nanoparticles aggregation, and it is expected that when larger sized nanoparticles aggregate, the transmission dip in their spectra widens far more than that of the smaller particles.

**Figure 4:**
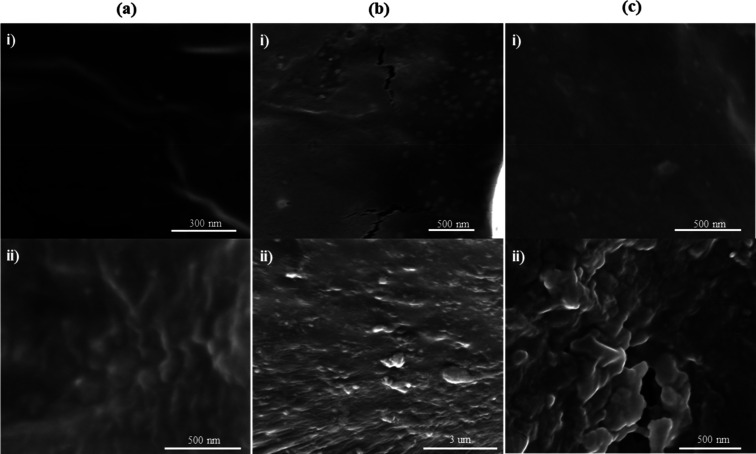
SEM micrographs of the (a) 12 nm GNCs, (b) 40 nm GNCs, and (c) 80 nm GNCs, where images (i) and (ii) refer to the lowest and highest concentrated samples denoted as A and D in Figure 3.

To verify the aggregation or cluster formation of the nanoparticles within the lens, SEM micrographs of the lowest and highest concentrated nanocomposites’ cross section were imaged, and they are shown in Figure 4. First, both the low and high concentrated 12 nm GNCs, shown in Figure 4a(i,ii), did not have noticeable aggregates, like the other two sets in Figure 4b,c. Also, the visible nanoparticles, which could be a bunch of clustered particles, were evenly dispersed. However, the size of the visible nanoparticles is slightly bigger in the highly concentrated sample of the 12 GNC than in the low concentrated sample. In fact, the average diameter of the nanoparticles in Figure 4a(i) was 74 nm, whereas that in Figure 4a(ii) was 136 nm, indicating that in the former an average of six particles were representative of a single particle (or aggregate), whereas in the latter the cluster was composed of 11 nanoparticles on average. Yet, this difference in the size of the NP clusters did not affect the fwhm of the transmission dip of the spectrum (Figure 3a), for the range of the fwhm shift was only 2 nm. Nonetheless, the clustering of the NPs changed the fwhm from its initial value at 34 nm (in water) to 52 nm.

On the other hand, differences in the distributions of the nanoparticles within the lenses were noticed for the 40 and 80 nm GNCs when their NP concentrations were varied (Figure 4b,c). For the 40 nm gold nanocomposite, the average diameters of the nanoparticles within the lowest and highest concentrated nanocomposites were 57 and 1455 nm, respectively. This indicates that in the lowest concentrated sample, shown in Figure 4b(i), almost all particles remained either unaggregated or formed clusters of less than three particles. Nonetheless, in the highest concentrated sample shown in Figure 4b(ii), some aggregates began forming, but unaggregated particles could also be noticed. The discrepancy between both samples was apparent in their SEM micrographs as well as in their transmission spectra (Figure 3a). The increase in the size of the particle aggregates caused the fwhm to increase from 64 to 80 nm. Similarly, NPs in the lowest concentrated sample of the 80 nm GNC formed fewer aggregates than those in the highest concentrated sample. Indeed, it is evident that most of the particles have been aggregated in Figure 4c(ii). For the transmission spectra of both 80 nm GNCs, the increase in fwhm can justifiably be attributed to the addition of the nanoparticles, which in fact caused further aggregation and formation of bigger clusters. Therefore, the increase in GNP concentration was detrimental in their aggregation state within the hydrogel. The formation of aggregates increased the average size of the nanoparticles and shifted the energy required to displace their electrons (decreased). This decrease in energy was reflected by an increase in wavelength; hence, the nanoparticles’ surface plasmon wavelength increased, and the transmission bandwidth increased due to the formation of unevenly dispersed particles. However, the nanocomposites in this case were only affected through their fwhm and not SPR wavelength, indicating that their aggregation state was not extremely severe.

**Figure 5:**
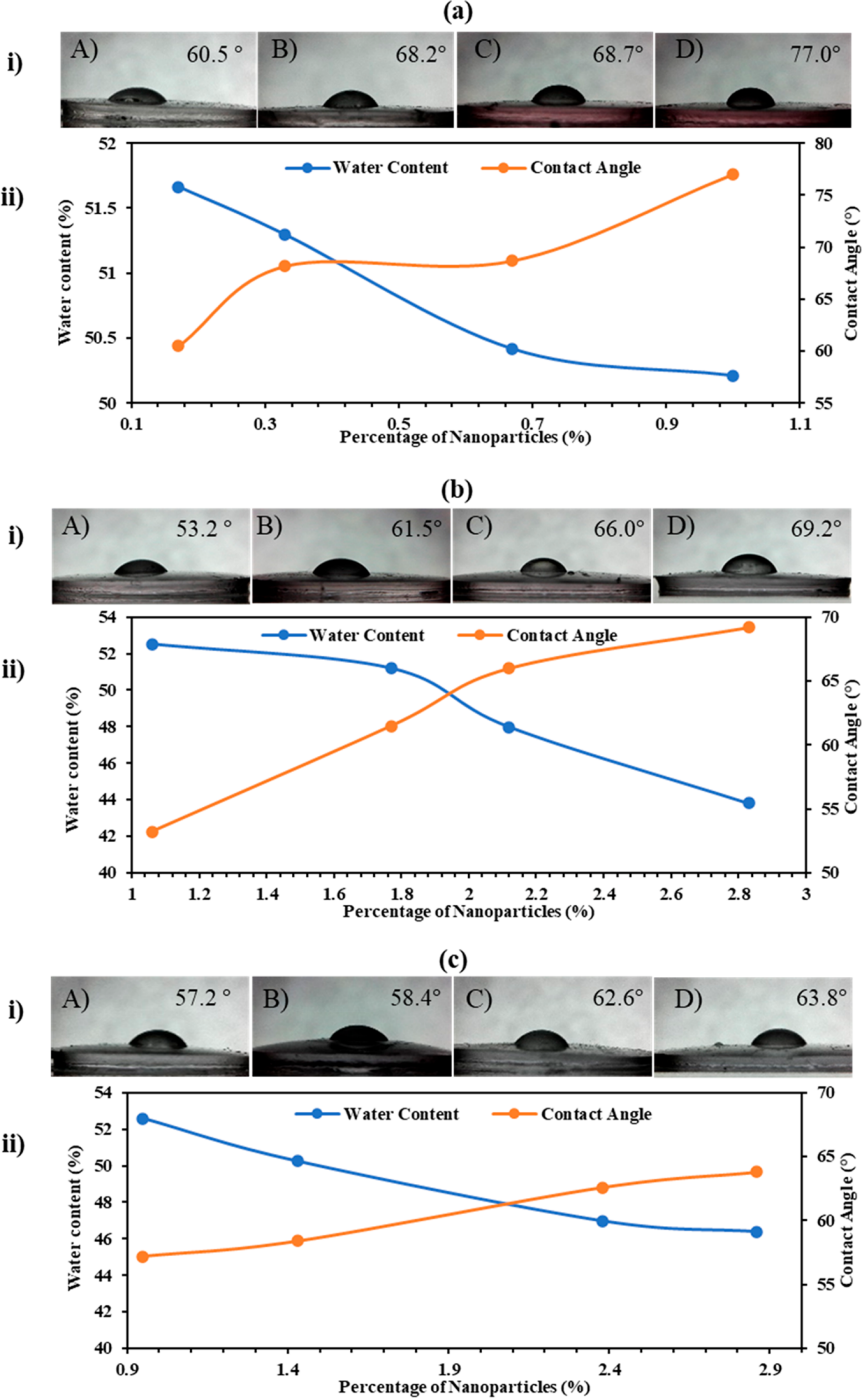
Wettability and water content measurements of the (a) 12 nm GNCs, (b) 40 nm GNCs, and (c) 80 nm GNCs: (i) contact angle measurements of the four nanocomposites, denoted as A–D in Figure 3, using the sessile drop method; (ii) effect of nanoparticle concentration on the water content and contact angle of the gold nanocomposites.

Figure 5 shows the effect of nanoparticle addition on the water content and contact angle of the three nanocomposite lenses. Generally, an analogous trend was observed in all the three plots of Figure 5 (ii). As expected, the water retention and wettability of the lenses decreased as a result of the increase in nanoparticles concentration. Thus, the surface of the contact lenses became more hydrophobic, and the swelling degree of the nanocomposites diminished.

Nonetheless, few quantitative discrepancies were present among the curves of the three sets. First, the range of contact angle shift was almost 17° for both the 12 and 40 nm GNCs, whereas that of the 80 nm GNC was 6.5°. The latter might have been due to the nanoparticles either not being abundant on the surface or them blending well within the polymeric chains. The latter is less possible as previous studies have shown that the incorporation of largely sized hydrophobic NPs (>70 nm) into the polymer results in the disruption of its chains on the surface.^34^ Despite the fact that the NP addition was at a constant rate, the effect on the contact angle was somewhat spontaneous. For instance, in the 12 nm GNCs, the contact angle initially increased by 7.7° and then was stagnant. The final addition of NPs caused the contact angle to rise by 8.8°. A more homogeneous trend was noticed in the water retention curve, which showed that the NP addition at each sample almost equally reduced the swelling ratios throughout. The increase in the NPs’ concentration reduced the water content of the three GNCs by 1.45, 8.7, and 6.2%, respectively. The small reduction in the water content of the 12 nm GNCs compared to that of the other two nanocomposite sets was due to them not forming large aggregates. However, for the 40 and 80 nm GNCs, the increase in nanoparticle concentration formed the visible aggregates as previously referred to in Figure 4b,c. The latter filled up the spaces between the polymeric chains and may have reduced the effective pore size; consequently, this reduced the ability of the hydrogel to retain water effectively. Yet, the reduction in water retention was less than 9%, which suggests that the aggregates did not severely hinder the transport within the hydrogel.

The general trend in all nanocomposites was similar: the increase in NP concentration caused a decrease in the water retention levels and the surface wettability of the lenses. This was expected as the hydrophobic nanoparticles could have blocked or slightly filled up some of the polymer chains’ pores. Nevertheless, discrepancies among the different sets did not follow a specific quantitative trend. It was expected that the size of the NPs would influence both properties, as it was previously reported that the largely sized NPs have higher influence on the water content and contact angle than the smaller ones.^34^ This was obtained only for one case: increasing the gold NPs size from 12 to 40 nm. The largest achieved water retention level was 52.5%, while the highest contact angle was 77°, indicating that the NPs did not cause the lens to be completely hydrophobic, and thus, they can be used in contact lens applications.

**Figure 6:**
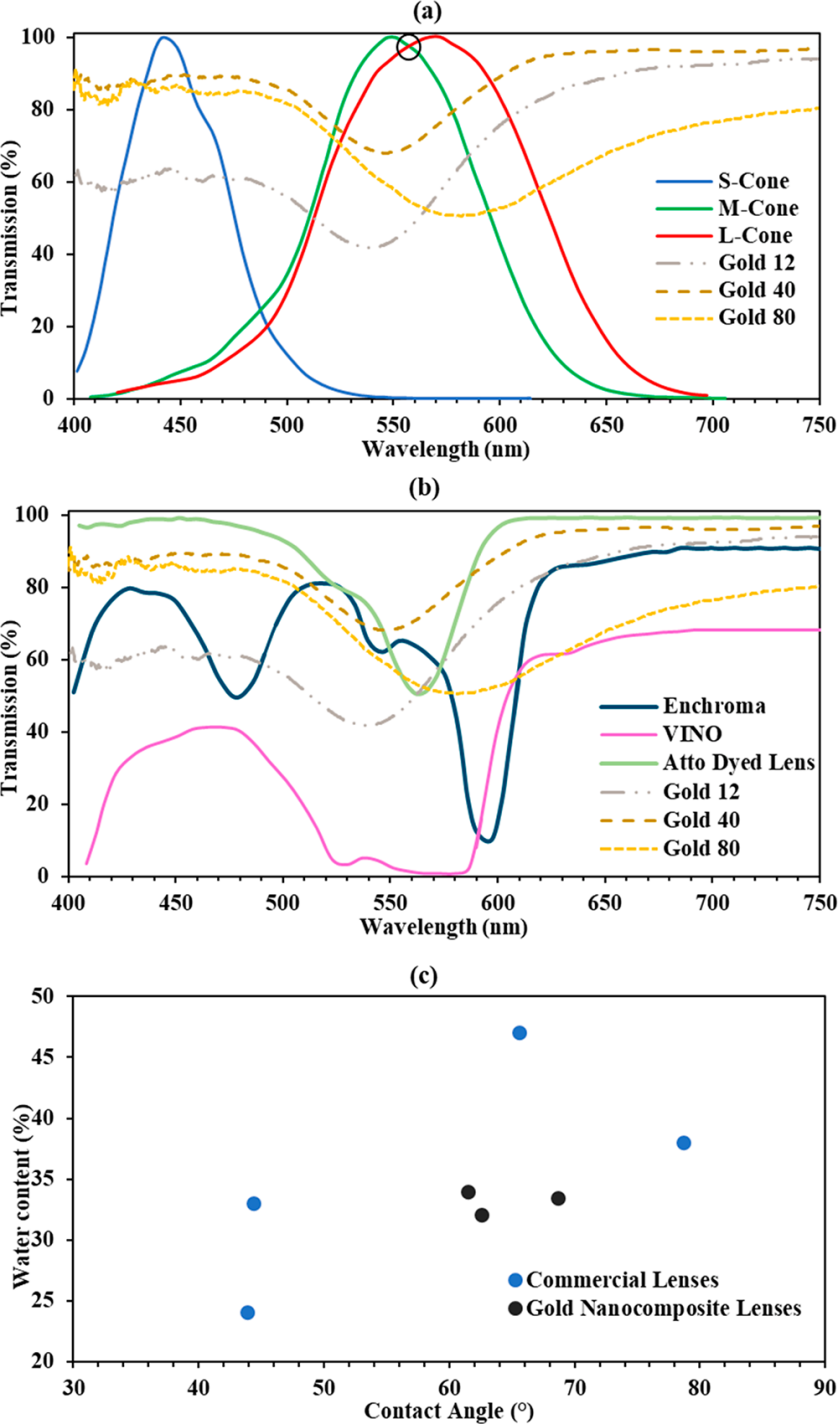
Performance evaluation of the nanocomposite lenses. (a) Transmission spectra of the 12, 40, and 80 nm gold nanocomposites in comparison to the spectral sensitivity of a protan’s or deutan’s photoreceptor cones. (b) Transmission spectra of the 12, 40, and 80 nm gold nanocomposites in comparison to the spectra of Enchroma, VINO, and the Atto dyed lens developed by.^22^ (c) Illustration of the contact angle and water content of some common commercial contact lenses in comparison to the developed nanocomposite lenses.

After characterizing the nanocomposites both through their optical and material properties, the performance of the developed lenses was evaluated against other CVD management wearables, and their efficacy as a suitable filtering technique was assessed. An optimum sample from each of the three sets of nanocomposites was chosen as the representative for that set (size). This was done based on the obtained lenses’ properties shown earlier. First, the effectiveness of the nanocomposites was evaluated by plotting their spectra along with that of a red–green CVD patient, and it is shown in Figure 6a. The deployed filter for a red–green CVD patient should block light at a specific wavelength in the spectrum, and this wavelength corresponds to the area at which both photoreceptor cells are activated simultaneously (intersection between both red and green curves). In Figure 6a, this intersection was circled in black, and the wavelength was found to be 560 nm. Moreover, the 12 nm gold nanocomposite’s transmission dip was 22 nm far from this intersection, yet it blocked 50% of light at that wavelength and was effective in transmitting the remaining wavelengths; the transmission was 80% beyond 605 nm. The 80 nm gold nanocomposite also blocked 47% of light at 560 nm, but it was not as effective as the 12 nm composite because up until 700 nm it transmitted only 75%, which is due to its broad transmission bandwidth. The most effective in filtering light was the 40 nm gold nanocomposite. Although it blocked only 31% of light at the intersection between both cones, its transmission rate was more than 90% beyond 600 nm. Also, its transmission was initially 88% as compared to that of the 12 nm nanocomposite, which was 62%. The issue of blocking more light can be bypassed by increasing and optimizing the concentration further so that the transmission bandwidth is not severely affected.

Furthermore, Figure 6b demonstrates the transmission spectra of the developed lenses compared to those of Enchroma, VINO, and an Atto dyed contact lens reported in ref (22). Enchroma and VINO glasses are some of the most widely used wearables by CVD patients, and their design and transmission blockage varies per the patient’s needs.^16^ The transmission dip of Enchroma was far from that of the Atto dyed lens, the 12 and 40 nm gold nanocomposites. Indeed, Enchroma’s transmission dip was more than 30 nm away from that of the latter mentioned lenses. Nonetheless, it was right in the region where the 80 nm gold nanocomposite lens blocked light, which was due to the wide transmission dip of the latter. For VINO, its transmission blocked light in 65 nm of the spectrum (from 520 to 585 nm); the transmission dips of all the developed lenses occurred at these wavelengths, indicating that VINO and the nanocomposites shared the same filtered wavelengths. The evident difference was that the developed gold nanocomposites were much more selective than VINO. Commercial products like Enchroma and VINO provide a variety of choices for CVD patients depending on the form of red–green deficiency from which they suffer. For the CVD wearables, the Atto dyed lens resembled the closest behavior both in SPR and fwhm to the gold nanocomposite lenses. In fact, the differences in SPR among the Atto dyed lens and the gold nanocomposites were 24, 15, and 11 nm, in respective order of their diameters; similarly, the fwhm differences were all less than 20 nm except for the 80 nm GNC, in which the difference was 71 nm. This indicates that if the Atto dyed lens was effective in wavelength filtering as proclaimed previously,^22^ the 12 and 40 nm gold nanocomposite lenses could also be similarly successful.

Finally, the discrepancies between the synthesized lenses and commercial ones in terms of their wettability and swelling ratio were studied and are shown in Figure 6c. It is worth mentioning that the method used to determine the contact angle is the sessile drop technique, in which a droplet of specific volume is placed on the lens, and the image of the droplet is recorded. Therefore, information on the contact angle of commercial lenses was collected from previous studies that utilized the same technique.^35,36^ As Figure 6c indicates, the hydration contact angle of the commercial contact lenses ranges between 44 and 79°, while their water content varies between 24 and 47%. Clearly, the moisture content and contact angle of all nanocomposites shown here were right in the middle of the aforementioned ranges and had wettability and water retention properties even better than those of some commercial lenses.

To sum up, the transmission of the synthesized 40 and 12 nm gold nanocomposite lenses filtered light effectively in regions where the red and green photoreceptor cones were overlapping. They also had transmission spectra very similar to that of the Atto dyed lens, which was proven effective through clinical trials. Their water content and contact angle attributes were comparable, and superior in few cases, to those of the commercial contact lenses. Finally, cytotoxicity analysis of the lenses was done using MTT reduction assay with Raw 264.7 cells as the model cells. The viability of the cells in both lenses was more than 75% after 24 h, indicating that all three optimum nanocomposite lenses were indeed biocompatible. Therefore, they can be used as wearable aids for deutans and protans.

## Conclusions

Color filtering contact lenses were successfully synthesized using gold nanoparticles with HEMA and EGDMA as a base polymer and a cross-linker, respectively. The size of the nanoparticles was determined using the TEM, and their transmission spectra were measured. Also, the effect of varying the medium refractive index of the particles on their transmission was analyzed. Moreover, three sets of GNPs with distinct sizes were utilized, namely, 12, 40, and 80 nm. Per each of the nanoparticle sets, four nanocomposites with varying concentrations were fabricated. Generally, the increase in NP concentration showed an increase in the transmission bandwidth, while the surface wettability and water content diminished. The optimum nanocomposites from each set of NPs were then selected, and their effectiveness as potential wearables for CVD patients was studied. The study showed that the transmission spectra of the 12 and 40 nm gold nanocomposite lenses were very comparable to those of the commercial and research-based CVD wearables. Further, the water retention and wettability properties of the fabricated nanocomposites were superior to a few of the commercially available contact lenses; thus, it was concluded that these lenses can be used to aid CVD patients. Results also showed that the nanocomposite lenses were biocompatible to macrophages. Finally, prior to deploying the nanocomposite lenses, their oxygen permeability should be measured. In fact, HEMA hydrogels have oxygen permeability lower than that of silicone-based hydrogels as the latter transmits oxygen directly through their siloxane group unlike HEMA hydrogels which absorb oxygen through water molecules. Therefore, after testing the efficacy of the GNCs in clinical trials, copolymerization of HEMA with a silicone-based hydrogel is vital to ensure high oxygen permeability of the lenses.

## Methods

### Preparation of Polymer Solution

The polymer utilized for contact lens fabrication was 2-hydroxyethyl methacrylate (HEMA), which was cross-linked with the ethylene glycol dimethacrylate (EGDMA), and 2-hydroxy-2-methylpropiophenone was used as an initiator. All polymers were purchased from Sigma-Aldrich and used as is without further purification. In the fabrication stage, HEMA, EDGMA, and the initiator were mixed with a ratio of 99:0.67:0.33, respectively. The polymer mixture was then added to a glass cuvette, which is left to sonicate for 30 min to ensure complete homogenization.

### Gold Nanocomposite Fabrication

**Figure 7:**
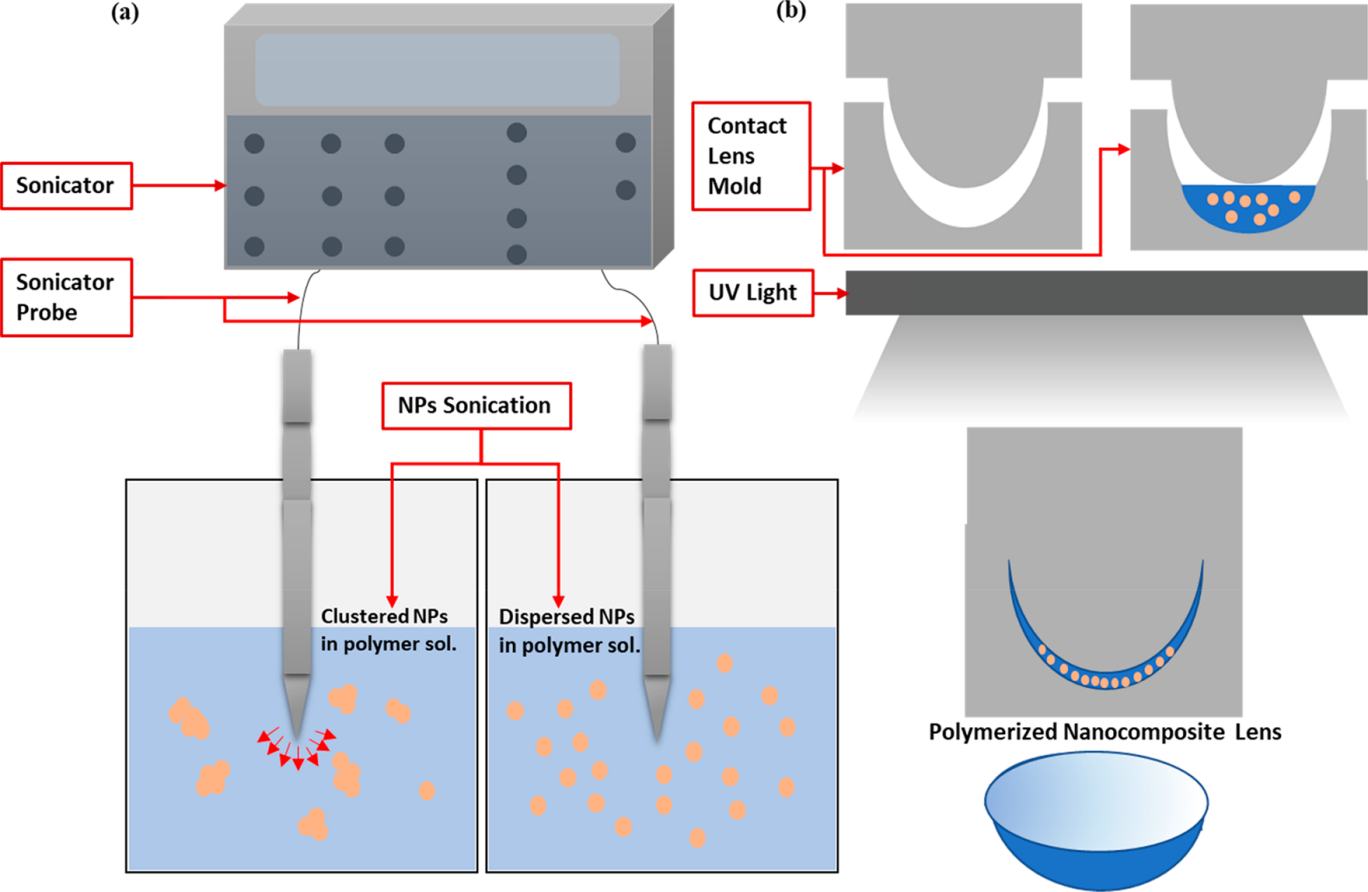
Schematic of the fabrication process of the nanocomposite contact lenses. (a) Ultrasonication of the nanoparticles to break initial agglomerates and clusters. (b) UV polymerization of the nanocomposite solution and formation of the nanocomposite lens.

In addition, gold nanoparticles with diameters of 10, 40, and 80 nm, stabilized in phosphate buffer solution with 0.1 mM concentration, were purchased from Sigma-Aldrich and used as is. The nanoparticle solutions to be tested were centrifuged to obtain a more concentrated form of them; 1 mL of the nanoparticles’ solution was centrifuged over three stages. The water in the resulting concentration was not completely dried to avoid irreversible aggregation of the particles. Then, depending on the required intensity of light absorption, a specific amount of nanoparticles was added to the polymer solution. Moreover, the polymer containing the nanoparticles was then sonicated for 30 min to ensure even distribution of the nanoparticles within the polymer and to breakup any aggregates that formed as a result of the centrifugation. After that, 110 μL of the solution was injected into the contact lens mold and left to polymerize under UV light for 5–10 min. The resulting hydrogel was washed twice using DI water to remove all of the residues. Figure 7 illustrates the steps carried out to make the hydrogel nanocomposite contact lenses.

### Gold Nanoparticles’ and Nanocomposites’ Characterization

The nanoparticles and nanocomposites were characterized before polymerization and postpolymerization. The prepolymerization characterization included obtaining the transmission/absorption spectrum of the nanoparticles, refractive index of the nanocomposite solution, and morphology characterization of the nanoparticles. The transmission spectrum of the nanoparticles was obtained using a USB 2000+ UV–vis spectrophotometer provided by Ocean Optics, which has a detection range of 400–1100 nm. Also, the refractive index of the nanoparticles’ medium was obtained using the KERN ORA-B. Fifty microliters of the solution was dispersed over the refractometer prism, and the measurements on the brix scale were recorded directly. Moreover, the brix value was then converted to the refractive index using the reference charts. Tecnai TEM 200 kV, which has a resolution of 0.24 nm, was used to characterize the morphology and size distribution of the nanoparticles. The voltage of the TEM can be varied from 20 to 200 kV. A few droplets from the nanoparticles’ colloidal solution were placed on the 300 mesh copper specimen grids purchased from Ted Pella. Ten microliters of the nanoparticles’ solution was added to the grid and was placed in a vacuum oven for 2 h at 50 °C to dry. This procedure was repeated three times to ensure a considerable number of nanoparticles stuck to the mesh grid. The details on the mean diameter of the nanoparticles and their size distribution were obtained using ImageJ software.

Moreover, like the prepolymerization characterization, in the postpolymerization characterization, the transmission spectrum of the nanocomposite was obtained, and SEM was used to study the distribution of the nanoparticles within the nanocomposite. In addition to the latter, contact lenses’ properties like water content and wettability were also obtained. The transmission spectrum was attained using the same UV–vis spectrophotometer (USB 2000+) discussed previously. After that, the nanocomposite was placed in a vacuum oven at 40 °C for 6 h, and its dry mass was recorded. Then, it was immersed and kept in deionized water for 72 h to ensure the maximum water retention possible. The swollen nanocomposite was then scaled, and the water content was obtained by deducting the dry mass from the total mass. In addition, the wettability of the contact lens was measured by obtaining its contact angle using the sessile drop method. Images were analyzed using ImageJ and the contact angle plug-in. Further, FEI Nova NanoSEM 650, which has an electron beam resolution of 0.8 nm, was used to examine the distribution of the nanoparticles within the nanocomposite lens. For SEM imaging, the nanocomposite was again placed in a vacuum oven for 6 h at 40 °C, after which it was dried and became hardened. It was then sheared using a cutting tool and coated with a 10 nm layer of palladium prior to imaging it through the SEM. The latter was done as the nanocomposite was charging when it was not coated.

Finally, the concentration of the nanoparticles was varied to study its effect on the nanocomposite lens, specifically the aforementioned properties. In fact, for each set of nanoparticles mentioned previously, four different concentrations were mixed with the polymer solution. The four NP concentrations were referred to as A, B, C, and D, where A and D had the lowest and highest concentrations, respectively. The nanocomposites were designed with names that identify their size and concentration. For instance, GNP12_A indicates that the nanocomposite was synthesized using 12 nm gold nanoparticles and had the lowest concentration in its set.
